# The Use of Weighted Graphs for Large-Scale Genome Analysis

**DOI:** 10.1371/journal.pone.0089618

**Published:** 2014-03-11

**Authors:** Fang Zhou, Hannu Toivonen, Ross D. King

**Affiliations:** 1 Division of Computer Science, The University of Nottingham, Ningbo, China; 2 Department of Computer Science, University of Helsinki, Finland; 3 Manchester Institute of Biotechnology, University of Manchester, Manchester, United Kingdom; Universitat Pompeu Fabra, Barcelona Research Park of Biomedicine (PRBB), Spain

## Abstract

There is an acute need for better tools to extract knowledge from the growing flood of sequence data. For example, thousands of complete genomes have been sequenced, and their metabolic networks inferred. Such data should enable a better understanding of evolution. However, most existing network analysis methods are based on pair-wise comparisons, and these do not scale to thousands of genomes. Here we propose the use of weighted graphs as a data structure to enable large-scale phylogenetic analysis of networks. We have developed three types of weighted graph for enzymes: taxonomic (these summarize phylogenetic importance), isoenzymatic (these summarize enzymatic variety/redundancy), and sequence-similarity (these summarize sequence conservation); and we applied these types of weighted graph to survey prokaryotic metabolism. To demonstrate the utility of this approach we have compared and contrasted the large-scale evolution of metabolism in *Archaea* and *Eubacteria*. Our results provide evidence for limits to the contingency of evolution.

## Introduction

Biology is undergoing a revolution due to the remarkable increase in the availability of DNA sequence data. This data is replete with biological knowledge. An increasingly important scientific challenge is therefore how best to extract this knowledge. This depends on the development of better data-structures and algorithms.

One area that has been transformed by the sequencing revolution is the area of prokaryotic metabolism. The complete genome sequences of over a thousand prokaryotic species are now known. These have been used to infer the compliment of enzymes available to these prokaryotic species, and hence their full metabolic competences — though of course caution must be used in interpreting such predictions.

Extracting knowledge from data requires use of some form of abstraction. The most natural and common abstraction for metabolism is that of graphs. In its simplest form a graph is a collection of *nodes* connected by *edges*. A number of different ways have been used to represent metabolic pathways using graphs (e.g. [1]–[3]). The representation we consider most useful is that of *labeled graphs*: where enzymes are abstracted to nodes, and the metabolites in reactions catalysed by these enzymes are abstracted to edges. A *labeled graph* is a graph where nodes or edges have labels: the nodes have unique enzyme labels, and the edges non-unique metabolite labels. Using this representation the whole metabolism of an organism may be represented as a large graph. Similar graph-based representations have been used in numerous studies to investigate the metabolism of different individual prokaryotic species (e.g. [4]).

**Figure 1:**
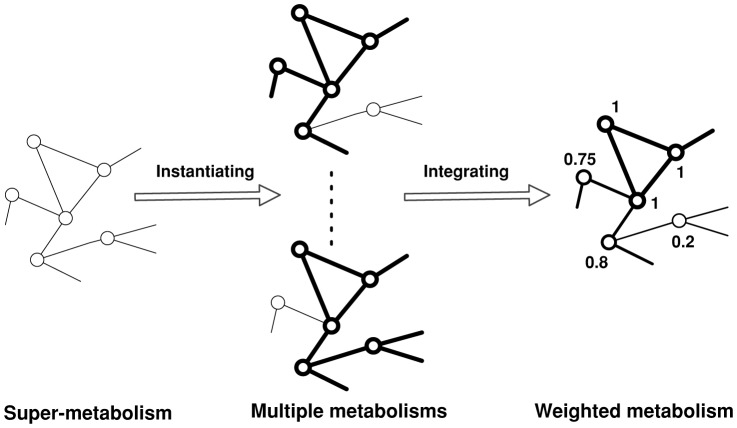
Constructing weighted metabolic networks. The metabolism of each species is a specific instantiation of the super-metabolism. Nodes in grey mean that they do not occur in a specific genome.

To compare the metabolisms from different species the most straightforward approach is to simply pairwise compare their metabolic graphs. Unfortunately this does not scale to the comparison of the metabolism of thousands of species. *We therefore propose the use of weighted graphs as a data structure for such large-scale analysis*. The idea is to integrate multiple metabolisms into one weighted network to simplify computation. A weighted graph is a graph where the nodes and/or edges have associated real numbers termed *weights*. For the case of weighted metabolic graphs we only consider weights on the nodes — enzymes. These weighted graphs are generated using a *super* metabolic graph. This is a graph with a node for every known enzyme in prokaryotic metabolism. The metabolic graph of each prokaryotic species may then be considered as a specific instantiation of this super-metabolic graph. An illustrated example is in Figure 1. The weights on the nodes summarize information from nodes in multiple genomes, for example, it could represent how common an enzyme is, or how similar enzyme sequences are.

### Surveying prokaryotic metabolism

A number of previous studies have surveyed the large-scale evolution of metabolism. Yamada & Bork [5] surveyed metabolic (and protein-protein) interactions using a graph-theory framework. In our view the most interesting surveys are those of Peregrin-Alvarez *et al.* [6] and Freilich *et al.* [7]. Peregrin-Alvarez *et al.* compared the metabolic graph of *E. Coli* with the genomic evidence known at the time. The main emphasis of the Freilich *et al.* paper is on comparisons with mammalian enzymes, compared to our work they also analysed an order of magnitude fewer genomes, and did not attempt to sample uniformly. More recently Kreimer *et al.* [8] examined 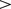
300 prokaryotic genomes using graph theory to estimate the *modularity* of metabolism. In an interesting application of large-scale analysis Borenstein *et al.* [9] estimated the environmental requirements of prokaryotic species.

### Three types of weighted graphs for surveying metabolism

We propose three different ways to use the instantiations of the super-metabolic graph to add weights to the nodes (enzymes) to form weighted graphs for the large-scale analysis of metabolic pathways.

The first approach is to define the enzyme weights to be the proportion of genome instantiations where a gene for the enzyme is found. We call this type of weighted graph *taxonomic*. Such graphs summarize the phylogenetic importance of enzymes: enzymes with high weights occur in many species, and those with low weights rarely occur.

The second approach is to define the enzyme weights to be the average number of protein sequences of that enzyme in a genome. We call these weighted graphs *isoenzymatic*. Such graphs summarize the enzymatic variety/redundancy of metabolism: a high number indicates more isoenzymes.

The third approach is to define the enzyme weights to be the average sequence similarity of that enzyme in the genome instantiations. These are termed *sequence similarity* weighted graphs. Such graphs summarize the sequence conservation of metabolism: a high number indicates high sequence conservation.

To the best of our knowledge weighted graphs have not been used in any of these ways before.

### Comparing the evolution of metabolism in *Eubacteria* and *Archaea*

We wished to test the utility of our weighted graphs for large-scale metabolic analysis. For this we selected the problem of investigating the diversity of prokaryotic metabolic pathways. Specifically we investigated the oldest and most fundamental split in the evolution of life, that between the two prokaryotic domains: *Archaea* and the *Eubacteria* [10]. Although recognition of the importance of this division came late [10], it is now clear that it is the deepest known phylogenetic division, and probably occurred 2–3 Billion years ago. Our idea is to use the newly available data on the biodiversity of metabolic pathways to investigate how different pathways have evolved since the divergence of the *Archaea* and the *Eubacteria*.

We recognize that because of gene transfer prokaryotic species/enzyme evolution does not have a pure tree topology (it is a directed acyclic graph). Genes have jumped between species, and across the *Archaea Eubacteria* divide. However, we hypothesize that this effect is insufficient to obscure the main signal from evolutionary descent.

### Sampling genomes

Any conclusions that we draw regarding prokaryotic metabolism should be generally true for prokaryotic genomes. However, only a limited number of genomes have been sequenced. If these sequenced genomes were an unbiased sample from all existing sequenced and non-sequenced prokaryotic genomes, then one could argue that the sample is representative of the whole. But unfortunately this is not the case. (1) The sequenced genomes are very biased towards prokaryotic groups that are of special interest to humans (e.g., pathogens), and also towards groups that are easy to cultivate in the lab. (2) When comparing *Eubacteria* and *Archaea* there is also the problem that *Eubacteria* genomes outnumber *Archaea* by an order of magnitude. It is unclear how much this imbalance is due to the fact that *Eubacteria* have been studied more (for example, because *Eubacteria* cause diseases while *Archaea* do not), and how much it is due to there being more species of *Eubacteria*. These biases mean that we had to be careful how we used the sequenced genomes. We therefore decided to sample genomes uniformly across evolutionary space. By this we mean that if you envisage prokaryotic species as leaves of the tree of life with the branches evolutionary distance, then we will sample leaves uniformly distant from each other.

## Materials and Methods

### Data preparation

To generate our super-metabolic graph we selected the 192 pathways that occur in prokaryota from Kyoto Encyclopedia of Genes and Genomes (KEGG) [11] (http://www.genome.jp/kegg/http://www.genome.jp/kegg/) (Release 59.0, July 1, 2011). This produced a graph with 2,365 enzymes with complete EC codes, and 43,627 metabolite edges (N.B. this is not the cardinality of the set of metabolites.).

We selected all prokaryotic (*Archaea* and *Eubacteria*) species with complete genomes from this release of KEGG. This gave 108 *Archaea* species and 1,287 *Eubacteria* species.

The assignment of protein function to these genes was taken directly from KEGG. It is clear that the functions of most genes from most genomes is not based on direct experimental evidence, but rather on inferred conservation of function with homology — a form of abductive reasoning [12]. Such inferences, like all abductions, are prone to error and must be treated with caution. However, these functional assignments are generally based on reasonably close homology, and are generally trusted. If these predictions were systematically wrong this could lead to bias in our results.

To sample genomes we first applied CD-HIT (http://weizhong-lab.ucsd.edu/cd-hit/) to cluster species based on their 16S ribosomal RNA sequences similarities at 0.8 level in each domain. We obtained 15 clusters of *Archaea* and 114 clusters of *Eubacteria* species. The different number of clusters reflect the difference in sampling (and possibly a difference in genomic diversity). To fairly compare sampling from the two domains, we sampled the same number of genomes from both domains. To generate the sampling datasets: for *Archaea* we randomly chose one species from each cluster; for *Eubacteria* we first randomly chose 15 clusters, then from each cluster randomly chose one species. We repeated the sampling process 100 times uniformly from both domains to provide 200 datasets each containing 15 genomes. We argue that this procedure produces datasets sampled uniformly across evolutionary space.

Our data is available free online at: http://www.cs.helsinki.fi/research/discovery/data/plosone2014/

### Weighted metabolic network construction

In a metabolic network 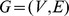, nodes correspond to enzymes: 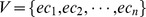. Two nodes (enzymes) 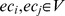 are connected, that is 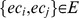, if the reactions which they catalyse share compounds. For example, consider the following two reactions where 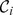 is a compound: (1) 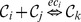, and (2) 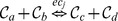. If the reactions share at least one compound, that is if 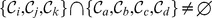, then 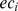 and 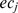 are connected by an edge. We here only take into account compounds whose entries begin with “C” in KEGG, and also remove run:common-cofactor-S1.pdfcommon cofactors (Table S1 (common cofactors)) taken from the article [13].

For taxonomic graphs let 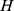 represent a set of selected genomes from one domain, 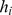 represent a genome in 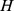, and 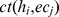 be a function showing whether 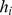 contains enzyme 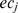. If 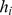 contains 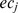, then 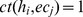, otherwise 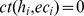. The taxonomic weight of an enzyme 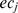 is 
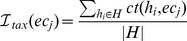
. The range of 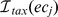 is [0,1]. A high taxonomic weight implies the enzyme ubiquitously exists in the domain.

For isoenzymatic graphs, the average isoenzymatic weight, denoted by 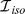, illustrates the average number of different protein sequences it may present. Let 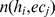 represent the number of different forms of protein sequences of an enzyme 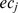 in the genome 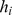. The isoenzymatic weight of an enzyme 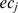 is
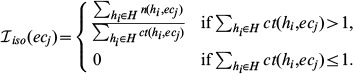
The range of 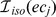 is 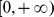. A high weight indicates a high redundancy of the enzyme.

For sequence similarity graphs we computed the average similarity of the enzyme's protein sequences, represented as 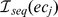, measuring how conserved an enzyme's protein sequence is during evolution. For each enzyme we first randomly picked one protein sequence of the enzyme from each selected genome, on the condition that the genome contains such enzyme. Let 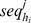 be the protein sequence of enzyme 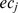 in the genome 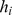. We then used the Laign [14] program of the FASTA package [15] with default parameters to compute the similarities of any pair of the selected protein sequences, denoted by 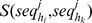. We next calculated the mean value of the pairwise similarities. The sequence similarity weight of an enzyme 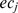 is
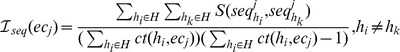
The range of 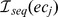 is [0,1]. A high sequence-similarity weight shows the enzyme's protein sequence conserved well.

For notational convenience, we write 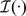 to represent either kind of weighting when we are not discussing a specific weighting.

### Inter and Intra domain correlation of weighted graphs

We wished to investigate the correlation between weights in the metabolic graphs. We first deleted non-informative regions of the graph: 1,112 enzymes have zero taxonomic weight in all 200 sampled datasets. (702 enzymes do not have taxonomy information in KEGG, and 347 enzymes only exist in *Eukaryota* kingdom, and the rest 63 enzymes exist in a tiny number of prokaryotic species.) For intra-domain correlations we included all enzymes that exist in at least two genomes in the selected data set. For the inter-domain correlations we examined all enzymes that have weights in either kingdom. We used Spearman's rank correlation to calculate correlations as it is robust. The correlations between datasets were calculated by using cor.test() function in the R program, and values of p
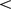
0.05 were considered to be significant.

We performed permutation tests to systematically examine the statistical significance of correlation between the weighted graphs we formed. In our setting the topology of the super-metabolic graphs in both domains is the same, and weights are only associated with nodes. Thus a rearrangement of the weights on the nodes produces a graph with the same topology and distribution of weights, but with no biological information — a random graph. We used these random graphs for the permutation tests. Let 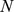 be the number of weighted enzymes in the graph. We randomly generated 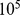 random graphs as a sample of the 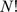 possible permutations. For illustration, let 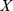 and 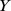 be two groups of enzyme weights. We first applied sample() function in the R program to rearrange the values of one group of data 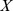 without replacement, denoted by 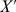, and then recomputed Spearman's rank correlation coefficient between the permuted group 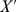 and the other group 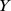. We repeated this step 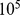 times, and totally obtained 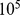 random correlation coefficient values. For the one-tailed test we calculated the ratio of the values in the permutational distribution of the statistic that are equal to or larger than the original correlation coefficient between 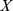 and 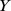. For ratios smaller than 0.05 we considered the null hypothesis not to be consistent with the observations.

### Specific or Ubiquitous enzymes across domains

We further analyzed the enzyme weights to identify whether the enzyme is specific or ubiquitous to a domain. An enzyme is regarded to be specific to a domain if its mean weight is above a specified high threshold in one domain, and is below a specified low threshold in the other domain. Conversely an enzyme is regarded as ubiquitous (to both domains) if its mean weights are above a specified high threshold in both domains.

Let 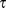 be the high threshold. For taxonomic weights and sequence-similarity weights, since both ranges are between 0 and 1, let 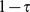 be the low threshold. When 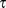 gets close to 1, then an enzyme is more specific to one domain. We set 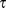 to be 0.667. For isoenzymatic weights, as the range is 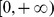, 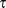 was set to be 1.

### Metabolic pathways

Traditionally sub-parts of metabolism have been classified into different *pathways*, and these pathways have proven useful in analysis. We therefore used pathway information from KEGG. Our construction of a weighted metabolic pathway 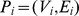 is analogous to the construction of the metabolic network. Node set 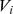 is the set of enzymes that are needed in the pathway 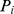. Two nodes (enzymes) are connected if the reactions which they catalyze are in the pathway 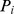 and also share compounds.

Because of limited availability of enzyme taxonomic data, nearly half of enzymes have zero weight in all sampled datasets. For analysis, we selected pathways that averagely contain at least 10 weighted enzymes in both domains. To examine the importance of each selected pathway to a domain, we calculated the ratio of specific (or ubiquitous) enzymes the pathway contains to the enzymes that averagely have non-zero weights in the pathway.

### Weighted graph compression

We further analyzed the weighted metabolic graphs using graph compression techniques. The model we applied is the extended version of the work, proposed by Toivonen et al [16], to take node weights into account during compression. Nodes with similar neighbors are grouped into super-nodes, and their edges grouped into super-edges. The idea is to compress an enzyme weighted metabolic network into a smaller one where the information related to enzymes with high weights is retained. We hypothesized that comparing compression across and between pathways would be informative for understanding the biodiversity and evolution of prokaryote metabolisms.

**Figure 2:**
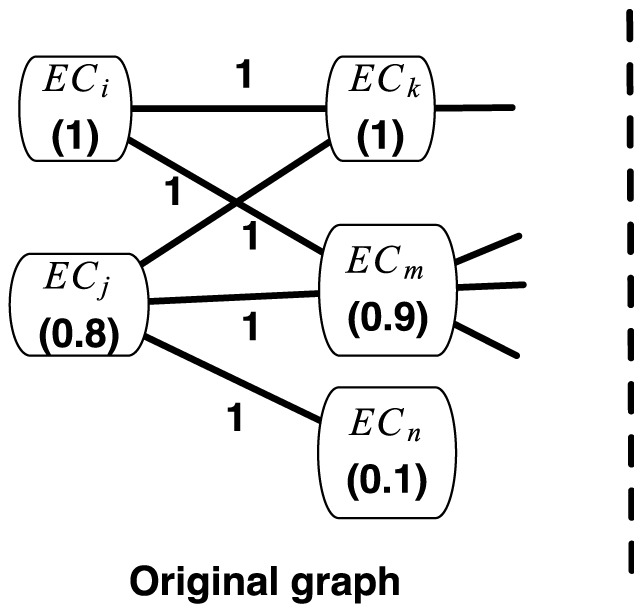
A example of weighted graph compression based on node weights. Node weights are in the parenthesis.

A graph is compressed iteratively through executing a series of operations. There are two basic operations: individual edge removal and node-pair merge. In the edge deletion operation, a single edge is removed, and if the removal leaves an edge endpoint isolated then the endpoint is removed as well. In the merge operation, a pair of nodes are merged into a new super-node, and the new super-node links with the neighbors of the merged nodes, and the weights on the super-edges re-assigned. Whether the new super-node links with their neighbors or not depends on whether the omission of the super-edge produces smaller error with respect to the extra saved space. In each iteration the effect of two operations are computed, and the one that produces a more compressed graph with smaller error is executed. An illustrated example is in Figure 2. Nodes 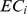 and 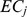 are merged into a new super-node. The new super-node only links with two previous neighbors 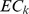 and 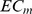, and the node 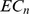 is deleted.

Compression may cause four types of error: a) a superedge may represent edges that do not exist in the original graph; b) edges in the original graph may not be represented by any superedge; c) nodes in the original graph may not be preserved in the compressed graph; and d) edge weights may be changed. The quality of the compressed graph is measured by computing the distance between the original graph and the decompressed graph of the compressed graph with respect to node weights, that is,
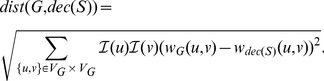
(1)Here 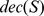 is the decompressed graph of the compressed graph 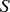, 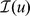 represents the weight on the node 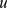, and 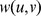 represents the weight on the edge between 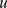 and 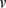. The decompressed graph 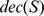 is a graph where nodes are all node identities that are inside the super-nodes in the compressed graph 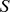, and edges link nodes if there are super-edges between the corresponding super-nodes. The weight of an edge equals the weight of the corresponding super-edge.

The weights on the superedges are set to minimize the distance between the original and the compressed graphs. However, the minimization problem is computational hard. Since the distance metric (Equation 1) satisfies the triangle inequality, the distance between the original and the compressed graphs is upper bounded by the sum of the distance between the increasingly compressed graphs. We then propose a solution to minimize the upper bound of the distance. The weights on the superedges therefore are set to minimize the distance between two sequential compressed graphs. Assume that (super)nodes 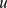 and 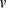 of graph 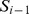 are compressed into supernode 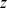 in the resulting graph 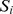, and 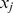 is one of 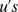 and 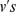 neighbors. The weight of the superedge 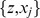 then is
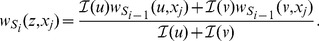
(2)The compression ratio 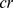 measures how much smaller the compressed graph is with respect to the original graph, which is defined as the cardinalities of nodes and edges, that is,
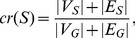
(3)where 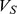 and 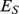 represent the number of super-nodes and super-edges in the compressed graph 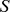, and 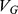 and 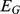 represent the number of nodes and edges in the original graph 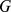. The values of 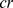 are in the range from 0 to 1. Specially, when 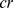 gets close to 0, the compressed graph becomes more abstract.

The goal of weighted graph compression is to produce a compressed graph 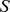 of a given weighted graph 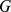 at a specified compression ratio 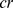, such that the distance between the original and compressed graph with respect to the node weights 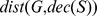 is minimized.

To investigate the conservation of evolution, we computed the average enzyme weights in the compressed graphs with respect to different compression ratios, and the average enzyme weights in the compressed part that exist in both *Archaea* and *Eubacteria* domains. We also calculated the similarity between the original and the compressed pathways. Suppose for the pathway 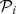, after compression, the number of removed nodes are 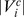, and the number of changed edges are 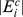 (including the number of added and deleted edges). The similarity between the original and compressed pathway is 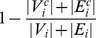. A large value indicates a high similarity between the original and the compressed pathway.

## Results

### The example of glycolysis

**Figure 3:**
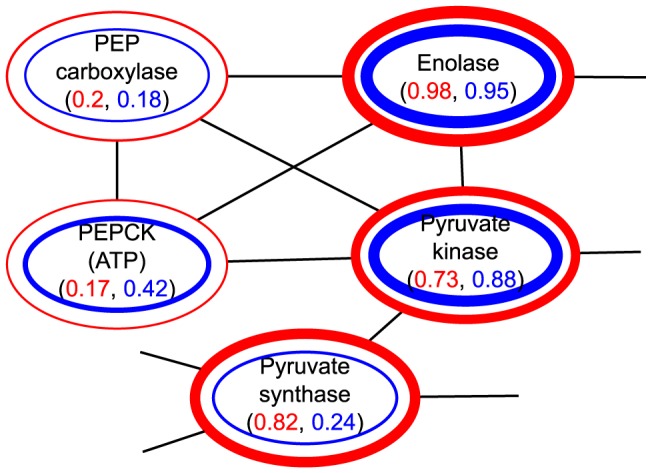
Taxonomic weight in a section of glycolysis. The weights for *Archaea* are in red, the weights for *Eubacteria* are in blue.

**Figure 4:**
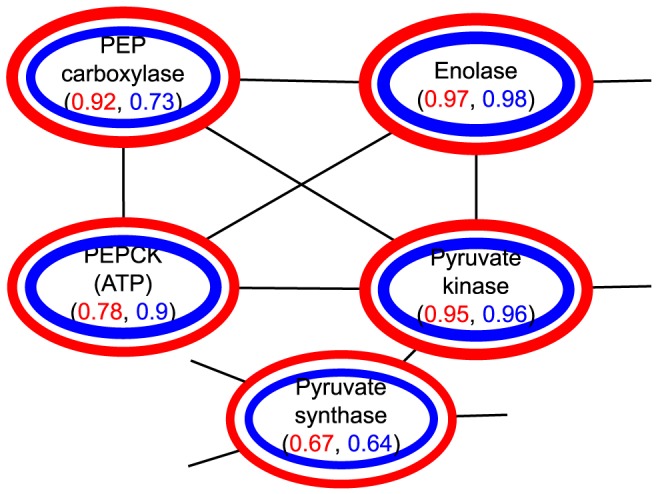
Sequence similarity weight in a section of glycolysis. The weights for *Archaea* are in red, the weights for *Eubacteria* are in blue.

**Figure 5:**
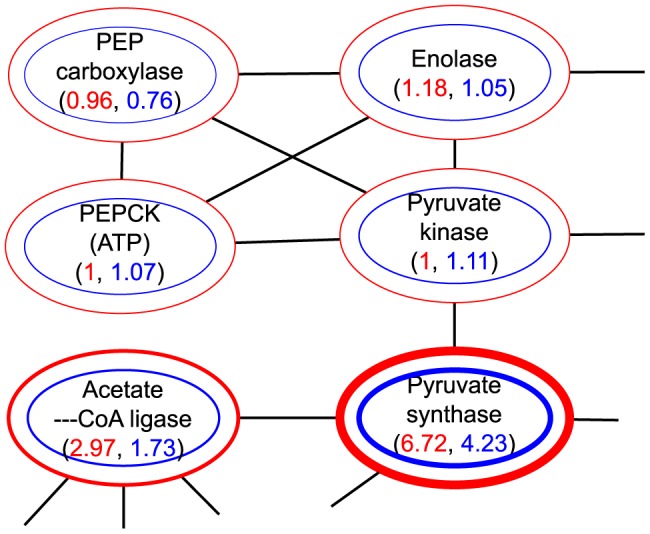
Isoenzymatic weight in a section of glycolysis. The weights for *Archaea* are in red, the weights for *Eubacteria* are in blue.

To illustrate the result of processing of the sampled metabolic networks into weighted graphs, Figures 3–5 show the result of forming the three types of weighted graph for a section of the glycolysis pathway for *Eubacteria* and *Archaea*. In order to better illustrate the differences of weights intuitively we have adjusted the thickness of nodes according to their node weights. We selected glycolysis because it is the proto-typical pathway, and because it is found in most prokaryota. Yet even in glycolysis large differences in weight are observed.

The taxonomic weights are in Figure 3. From the figure it is clear that some enzymes are ubiquitous, i.e. found in most species of both *Eubacteria* and *Archaea*, e.g. pyruvate kinase (EC 2.7.1.40); while other enzymes occur more frequently in one domain or the other, e.g. pyruvate synthase (EC 1.2.7.1) occurs much more frequently in *Archaea* than *Eubacteria*; other enzymes are uncommon in both domains, e.g. PEP carboxylase (EC 4.1.1.32).

The sequence similarity weights are in Figure 4. Here again there are large differences in weights across the pathway and between *Eubacteria* and *Archaea*. Enolase (EC 4.2.1.11) has relatively high sequence similarity in both *Eubacteria* and *Archaea*, while Pyruvate synthase (EC 1.2.7.1) has relatively low sequence similarity. In contrast PEP carboxylase (EC 4.1.1.32) has a high sequence similarity in *Archaea* and low similarity in *Eubacteria*. Such differences do not seem to be explainable by technical problems with alignment or sampling.

The isoenzymatic weights are in Figure 5. There are sizable differences in this type of weights across the pathway and between *Eubacteria* and *Archaea*. PEP carboxylase (EC 4.1.1.32) has the lowest isoenzymatic weights in both *Eubacteria* and *Archaea*, while Pyruvate synthase (EC 1.2.7.1) has the highest weights. The most notable difference between *Eubacteria* and *Archaea* is Acetate—CoA ligase (EC 6.2.1.1).

### The relationship between the different types of enzyme weights

**Figure 6:**
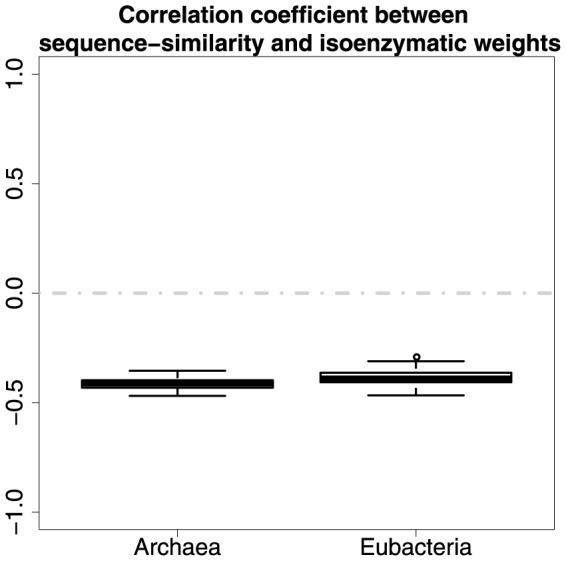
Correlation between sequence similarity and isoenzymatic weights.

**Figure 7:**
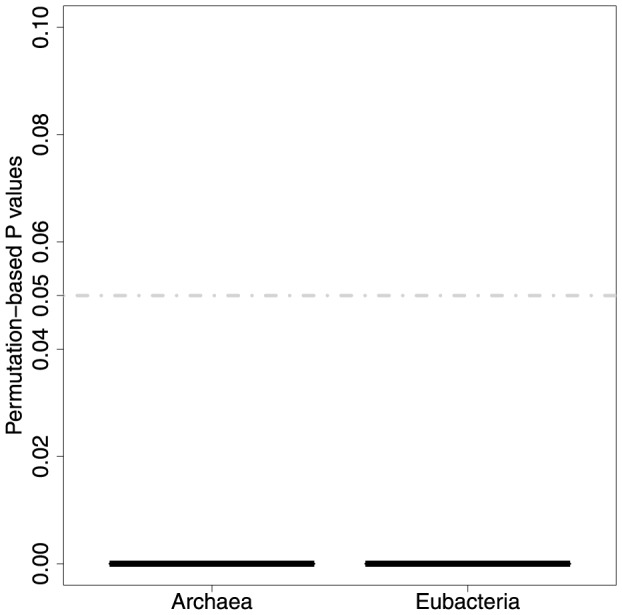
The strength of relationship between sequence similarity and isoenzymatic weights as estimated by permutation tests.

We investigated the correlation relationship between the different types of enzyme weights. In both *Archaea* and *Eubacteria* there is a statistically significant and moderate negative correlation between sequence-similarity and isoenzymatic weights (Figure 6). The average correlation coefficient values are 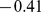 and 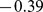 respectively. The average P values (of 100 tests) for these significance values are 4.18e-16 and 4.31e-15. The average permutation-based P values (Figure 7) in both *Archaea* and *Eubacteria* are 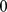, which show that there is a highly significant relationship between sequence-similarity and isoenzymatic weights in both domains. This means that enzymes where the sequences have low sequence similarities are more likely to have a greater number of isoenzymes, this seems intuitively reasonable as isoenzymes could enable greater sequence divergence.

**Figure 8:**
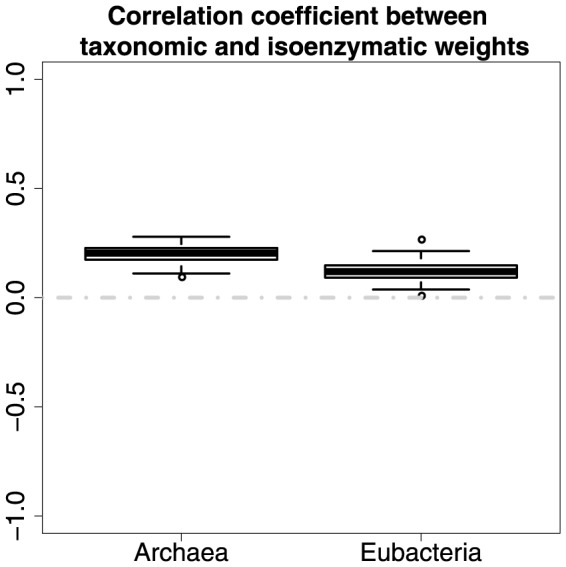
Correlation between taxonomic and isoenzymatic weights.

**Figure 9:**
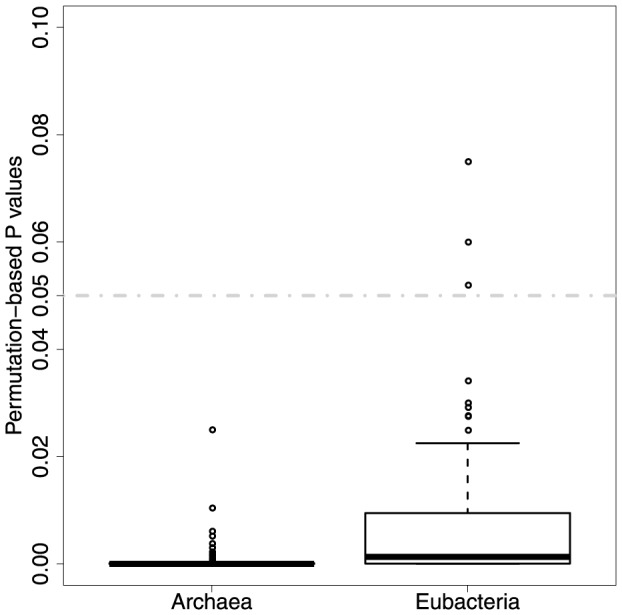
The strength of relationship between taxonomic and isoenzymatic weights as estimated by permutation tests.

In *Archaea* there is a weak but statistically significant positive correlation (Correlation coefficient = 0.2, P value = 0.0013) between taxonomic and isoenzymatic weights (Figure 8). This suggests that more common enzymes are slightly more likely to have isoenzymes. This also seems reasonable, as the more common enzymes are likely to be more important and need greater control, but it is unclear why the correlation is so low. In *Eubacteria* the correlation between taxonomic and isoenzymatic weights is negligible (Correlation coefficient = 0.12, P value = 0.035). Their significance test results (Figure 9) indicate that a clear relationship (average permutation-based P value = 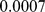) between taxonomic and isoenzymatic weights exists in Archaea, whereas the relationship (average permutation-based P value = 0.017) is not quite obvious in Eubacteria. It is not clear why there is a difference between domains.

**Figure 10:**
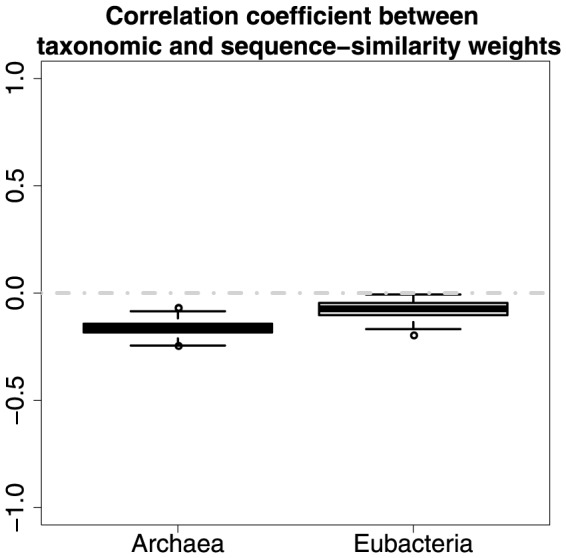
Correlation between taxonomic and sequence similarity weights.

There is a weak but statistically significant negative correlation (Correlation coefficient = 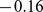, P value = 0.008) between taxonomic and sequence-similarity weights in *Archaea* (Figure 10). This means that more common enzymes are slightly more likely to have less diverged sequences. This is also reasonable, as they are likely to be under more constraints. The corresponding negligible negative correlation (Correlation coefficient = 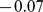, P value = 0.18) in *Eubacteria* is not significant. The significance tests (Figurel 11, average permutation-based P values of 0.0038 and 0.09 respectively) give the same outcomes. Again it is not clear why there is a difference between domains.

**Figure 11:**
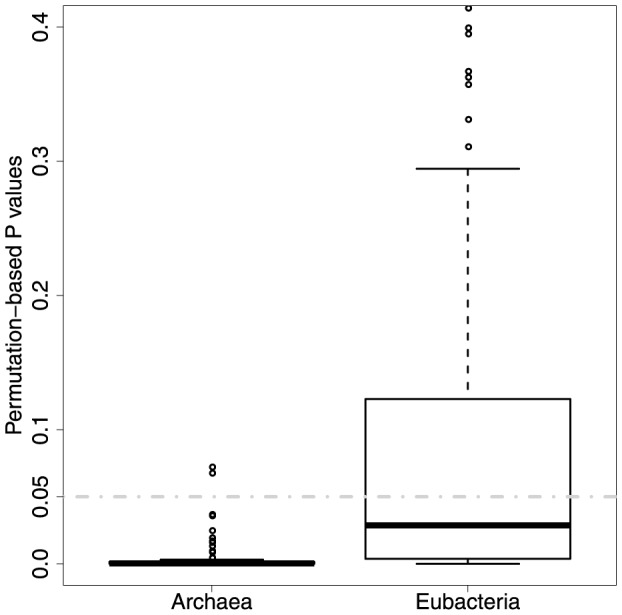
The strength of relationship between taxonomic and sequence similarity weights as estimated by permutation tests.

It is noteworthy that in all cases the correlation is stronger in *Archaea* than *Eubacteria*, the reasons for this are unclear.

### Comparison of the *Archaea* and *Eubacteria* domains

#### Summary statistics

We calculated the mean weights for all enzymes, and for all enzymes with non-zero weights for the three weightings (Table 1). The mean weights for *Archaea* are set in roman, and mean weights for *Eubacteria* are in italics. The mean weights for a specific type of weighting are similar across domains.

**Table 1:** Mean enzyme weights in different types of weights. The weights for *Archaea* are set in roman, the weights for *Eubacteria* are in italics.

	All enzymes		Non-zero enzymes	
	Archeae	Eubacteria	Archaea	Eubacteria
Taxonomic weight 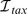	0.104	*0.14*	0.334	*0.271*
Isoenzymatic weight 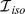	0.257	*0.367*	1.06	*0.868*
Sequence-similarity weight 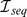	0.168	*0.247*	0.694	*0.58*

**Figure 12:**
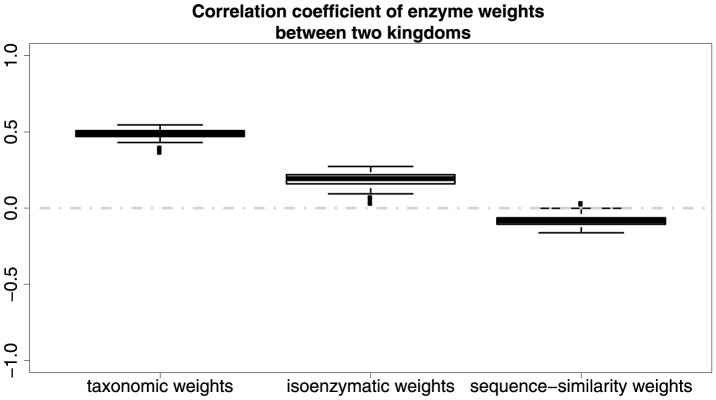
Correlation of weights between domains.

**Figure 13:**
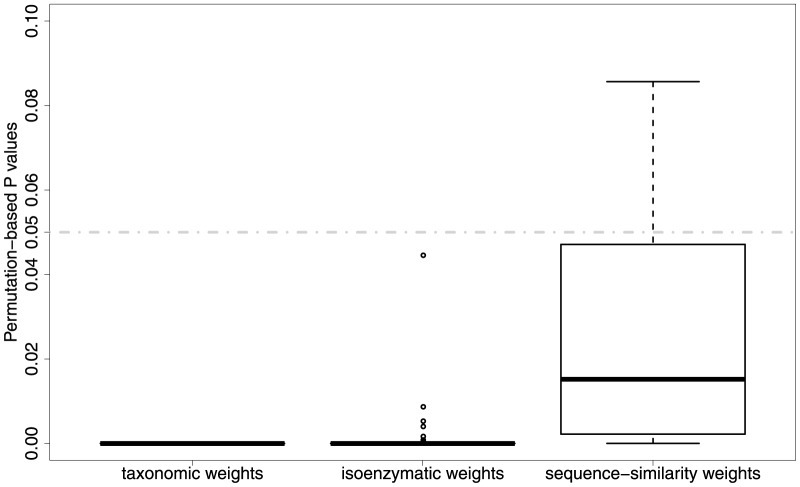
The strength of relationship of weights between domains as estimated by permutation tests.

We then used Spearman's rank correlation to compute correlation of the weights as this gives a broad indication how similar the metabolic graphs are across domains (Figure 12). The correlation of 0.48 in taxonomic weights between domains (i.e. *Archaea* and *Eubacteria*) is highly significant (P value = 1.468e-28). The correlation of 0.19 in isoenzymatic weights between domains is also significant but at a much lower level (P value = 0.0055). There is however no significant correlation (Correlation coefficient = 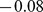) in sequence-similarity weights between domains in *Archaea* and *Eubacteria* (P value = 0.124). The significance test results (Figure 13) provide evidence that average permutation-based P values are 0 and 0.0027 respectively, so a clear relationship exists between domains in both taxonomic weights and isoenzymatic weights, but the relationship (average permutation-based P value = 0.067) between domains is not quite obvious in sequence-similarity weights.

#### Individual enzymes

In order to compare the similarities and differences between *Archaea* and *Eubacteria* in more detail we identified specific and ubiquitous enzymes across domains.

In the case of taxonomic weights, for the 1,253 enzymes that have weights in either kingdom, 73 enzymes are classed as domain specific, and 120 as ubiquitous. For example valine-tRNA ligase (EC 6.1.1.9) occurs in every genome examined. (Valine is in some sense the most average of amino acids.) The commonest enzyme that isn't associated directly with macromolecule processing is ribose-phosphate diphosphokinase (EC 2.7.6.1). Other extremely ubiquitous enzymes are phosphoglycerate kinase (EC 2.7.2.3) and glycine hydroxymethyl-transferase (EC 2.1.2.1).

Some enzymes occur much more frequently in one domain than the other. Some of these enzymes are expected, for example UDP-N-acetylmuramate dehydrogenase (EC 1.1.1.158) is involved in peptoglycan metabolism and is much more common in *Eubacteria*. However, others are more surprising, such as the comparatively frequent occurrence of L-lactate dehydrogenase (EC 1.1.1.27) in *Eubacteria*, but not *Archaea*. Examples of enzymes that occur much more frequently in *Archaea* are mevalonate kinase (EC 2.7.1.36), and nicotinamide-nucleotide adenylyltransferase (EC 2.7.7.1).

#### Pathways

To examine the importance of each pathway to the domain we calculated the ratio of special enzymes (e.g. specific or ubiquitous enzymes) to the size of enzymes with non-zero weights in each pathway (Table 2). The pathways that contain the highest ratio of ubiquitous enzymes are: aminoacyl-tRNA biosynthesis; valine, leucine, and isoleucine biosynthesis; and pheylalanine, tyrosine, and tryptophan biosynthesis. The origin of these pathways clearly predates the *Eubacteria Archaea* divide, and we may speculate they are perhaps the oldest of all pathways. The pathways that contain the highest ratio of specific enzymes are: terpenoid backbone biosynthesis, and pantothenate and Co-A biosynthesis. It is also noteworthy that both glycolysis/gluconeogenesis has quite a high ratio of specific enzymes.

**Table 2:** The ratio of specific or ubiquitous enzymes to the enzymes that have weights in the pathways. 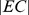 is the number of enzymes that have weights.

Pathway Name	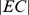	Taxonomy	
		Specific	Ubiquitous
Aminoacyl-tRNA biosynthesis	31	0.03	0.645
Valine, leucine and isoleucine biosynthesis	16	0	0.625
Phenylalanine, tyrosine and tryptophan biosynthesis	24	0.08	0.54
Pantothenate and CoA biosynthesis	18	0.22	0.33
Carbon fixation in photosynthetic organisms	23	0.087	0.304
Pyrimidine metabolism	46	0.02	0.3
Alanine, aspartate and glutamate metabolism	33	0	0.3
Purine metabolism	72	0.083	0.25
Histidine metabolism	22	0	0.227
Glycolysis/Gluconeogenesis	44	0.09	0.205
Glycine, serine and threonine metabolism	46	0	0.174
Lysine biosynthesis	24	0.08	0.17
Pentose phosphate pathway	31	0.097	0.16
Terpenoid backbone biosynthesis	25	0.4	0.16
Citrate cycle (TCA cycle)	20	0.05	0.15
Cysteine and methionine metabolism	42	0.05	0.14
Selenocompound metabolism	15	0	0.13
Methane metabolism	56	0.107	0.107
Arginine and proline metabolism	76	0.026	0.079
Fructose and mannose metabolism	40	0.025	0.075
Amino sugar and nucleotide sugar metabolism	55	0.036	0.072
Pyruvate metabolism	46	0.087	0.065
Nicotinate and nicotinamide metabolism	16	0.125	0.0625
One carbon pool by folate	17	0.112	0.059
Carbon fixation pathways in prokaryotes	36	0.11	0.06
Butanoate metabolism	38	0.026	0.053
Glutathione metabolism	21	0	0.048
Porphyrin and chlorophyll metabolism	52	0.019	0.04
Galactose metabolism	26	0.038	0.038
Valine, leucine and isoleucine degradation	27	0	0.037
Nitrogen metabolism	41	0	0.024
Starch and sucrose metabolism	50	0	0.02
Propanoate metabolism	31	0.1	0
Glyoxylate and dicarboxylate metabolism	38	0	0
Pentose and glucuronate interconversions	38	0.079	0

#### Compression results on average enzyme weights

To further investigate the conservation of evolution we applied weighted graph compression to abstract the metabolic networks into smaller ones utilizing taxonomic enzyme weights. The idea of using compression is that with increased compression more and more nodes and edges with lower weights are removed; therefore the compressed graphs may also be informative about the common ancestor of *Eubacteria* and *Archaea*.

**Figure 14:**
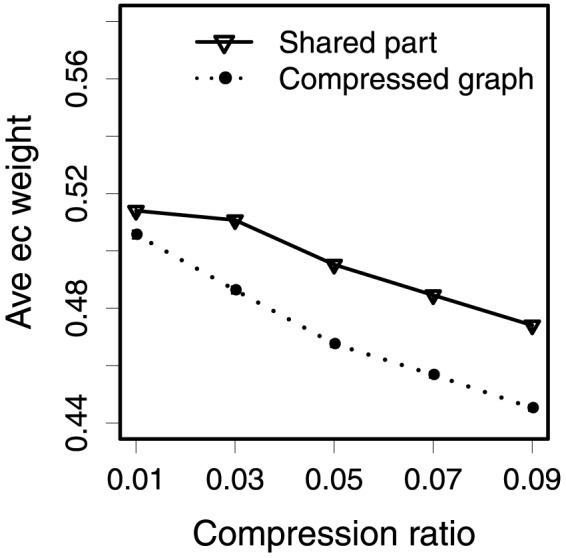
Average weight of enzymes in the compressed Archaea graphs, and average importance of enzymes in the compressed part shared by two domains.

**Figure 15:**
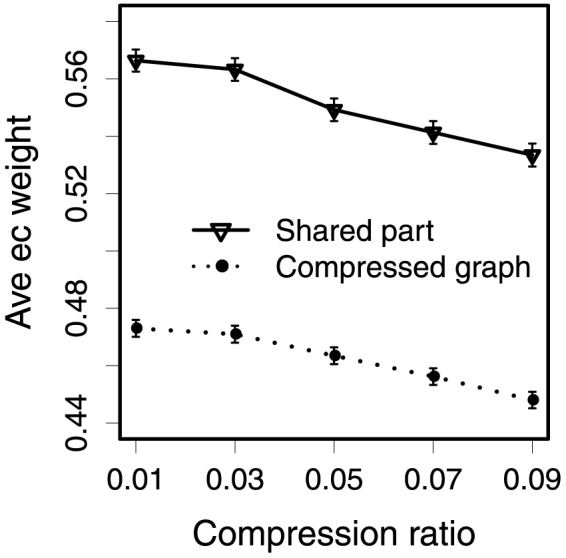
Average weight of enzymes in the compressed Eubacteria graphs, and average importance of enzymes in the compressed part shared by two domains.

We calculated the average taxonomic enzyme weight in the compressed graphs as a function of compression ratio, and plotted the results of *Archaea* in the dashed-line in Figure 14, and the results of *Eubacteria* in Figure 15. As expected, the average enzyme weight increases when there is more compression, i.e., in a smaller compression ratio. As enzymes with relatively lower weights are removed in the compression process, the enzymes left in the compression graphs become more important to the kingdom. We next computed the average taxonomic weight of enzymes in compressed part shared by both kingdoms. The results, plotted with solid lines in Figures 14 and 15, show that the average taxonomic weight of enzymes in the shared compressed part also becomes higher when there is more compression. More interestingly, the average weight in the shared part for a specific kingdom (e.g., Figure 15) is much higher than the weight in the whole compressed graphs.

#### Compression results on pathways

We further investigated how similar the compressed pathways are to the original ones. The correlations of compression results of pathways across domains are significant and very strong positive in three types of weights (Table 3).

**Table 3:** The correlations of compression results between domains.

	Correlation	P value
Taxonomic weight 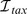	0.91	3.91e-14
Isoenzymatic weight 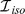	0.83	8.27e-10
Sequence-similarity weight 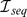	0.82	1.65e-09

We ranked pathways based on the descending similarity between the original and the compressed graphs (Table 4). The pathways in the top rank have high similar original structure in the compressed results. Since the average enzyme weight becomes higher when there is more compression (Figures 14 and 15), the pathways in the top rank probably contain more enzymes with high weights, and it also implies that parts of these pathways possibly exist in the ancestor of the domain.

**Table 4:** The rankings of pathways with respect to the descending similarity between the original and the compressed pathways (compression ratio is 0.09).

Pathway Name	Taxonomy		Isoenzyme		Sequence-similarity	
	Archaea	Eubacteria	Archaea	Eubacteria	Archaea	Eubacteria
Aminoacyl-tRNA biosynthesis	1	4	4	3	4	3
Selenocompound metabolism	2	10	14	11	14	9
Citrate cycle (TCA cycle)	3	1	3	1	3	1
Valine, leucine and isoleucine biosynthesis	4	2	2	2	2	2
Carbon fixation in photosynthetic organisms	5	7	5	10	5	11
One carbon pool by folate	6	8	9	6	9	6
Pentose phosphate pathway	7	5	15	4	15	4
Porphyrin and chlorophyll metabolism	8	9	6	8	6	8
Carbon fixation pathways in prokaryotes	9	3	11	5	11	7
Glycolysis/Gluconeogenesis	10	15	8	17	8	10
Alanine, aspartate and glutamate metabolism	11	11	13	9	13	10
Lysine biosynthesis	12	6	10	7	10	5
Methane metabolism	13	24	12	26	12	27
Phenylalanine, tyrosine and tryptophan biosynthesis	14	14	7	16	7	16
Pyrimidine metabolism	15	16	16	13	16	13
Glycine, serine and threonine metabolism	16	21	18	21	18	21
Pantothenate and CoA biosynthesis	17	17	17	18	17	14
Pyruvate metabolism	18	19	28	20	28	20
Nitrogen metabolism	19	12	22	12	22	12
Terpenoid backbone biosynthesis	20	13	1	14	1	15
Butanoate metabolism	21	20	26	19	26	19
Histidine metabolism	22	26	21	27	21	26
Cysteine and methionine metabolism	23	25	23	22	23	22
Arginine and proline metabolism	24	22	24	24	24	23
Amino sugar and nucleotide sugar metabolism	25	23	25	23	25	25
Propanoate metabolism	26	35	27	32	27	32
Nicotinate and nicotinamide metabolism	27	28	20	29	20	28
Purine metabolism	28	18	19	15	19	17
Fructose and mannose metabolism	29	27	31	28	31	29
Valine, leucine and isoleucine degradation	30	29	30	25	30	24
Galactose metabolism	31	30	29	30	29	31
Glyoxylate and dicarboxylate metabolism	32	31	32	34	32	33
Starch and sucrose metabolism	33	34	33	35	33	35
Pentose and glucuronate interconversions	34	33	34	31	34	33
Glutathione metabolism	35	32	35	33	35	34

## Discussion

### Summary of prokaryotic metabolism

The weighted graphs, in Figures 3–5, concisely and intuitively illustrate how different enzymes exist between the prokaryotic domains. According to Figure 12, for taxonomic weights, there is moderate correlation (0.48) between two domains. This implies that if an enzyme is common in species in one domain, it is likely to also be common in the other domain. In contrast the correlation (0.19) between the number of isoenzymes for a given enzyme is weak, and there is no correlation (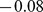) in sequence-similarity weights.

By comparing different types of weighted graphs we can also get some useful understandings of the correlation among sequence-similarity weights, isoenzymatic weights and taxonomic weights. For example, when an enzyme is common in a domain it is more likely to have a higher number of isoenzymes (Figure 6).

Another useful analysis is about how many specific or ubiquitous enzymes each pathway contains (Table 2). If a pathway contains a high ratio of ubiquitous enzymes it is more likely that the pathway exists in the core metabolism of both domains. In contrast, a pathway contains a high ratio of specific enzymes implies that the pathway is more common in one domain.

### The utility of weighted graphs in analyzing the contingency of evolution

One of the most fundamental questions in evolutionary biology is to what extent the paths that evolution has taken are stochastic, and to what extent they are determined by constraints imposed by the environment and biochemistry. Eminent evolutionary biologists have taken radically different views on this question of stochasticity. Stephen Jay Gould in many essays, and most notably in his book *Wonderful Life* [17], argued for contingency in evolution. For him evolutionary biology, in seeking to explain the past was a historical science, so if the process could somehow be run again then one would expect a radically different result. In contrast Simon Conway Morris [18] has argued that the constraints on living organisms are such that it is likely that evolution would take broadly the same path and intelligent organisms such as humans are likely to evolve.

The central problem with investigating this question is that it is generally impossible to repeat the experiment — evolution. However, it is possible to get some understanding of the stochasticity of the problem by looking at cases where evolution started from the same starting points, i.e. the *Archaea* and *Eubacteria*.

Our most relevant results regarding this question are Figure 12 and Table 4. In Figure 12 the correlation of 0.48 between taxonomic weights in *Archaea* and *Eubacteria* is highly significant (P value = 1.468e-28). This means that knowing the phylogenetic importance of an enzyme in one domain is informative about its importance in another domain. Our interpretation of this result is that as the process of evolution proceeded and new species of prokaryota were formed, evolution was constrained to use enzymes similarly in both domains. We argue that these results are evidence for limits to the contingency of evolution, however it is difficult to quantify the extent of contingency.

A similar but weaker pattern is seen for isoenzymatic weights. However, there is no correlation in the case of sequence-similarity weights. Our interpretation of this is that most sequence changes are neutral and not selective [19]. If this is the case then we would expect to observe little correlation between domains for this type of weight.

Figures 14 and 15 show, for a specific domain, the average taxonomic enzyme weight in the compressed part shared by both domains is higher than the weight in the whole compressed graph. This means the enzymes in the shared part have high weights in both domains. Our interpretation of this is that the shared enzymes are more common than average in both kingdoms. These results provide the evidence of the conservation of evolution. Likewise, Table 4 lists the ordered pathways that are well preserved in the compressed graphs, which indicates that parts of these pathways are more likely to have been present in their common ancestral organism.

The correlation of compression results of pathways between domains (Table 4) is statistically significant and very strong positive (Correlation coefficient = 0.91, P value = 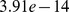) for taxonomic weights. This means the pathways that are important in one domain are also important in another domain. Again this is evidence of limits to the contingency of evolution.

### Application of weighted graphs to other biological networks

We have demonstrated the use of weighted graphs as a way of efficiently analyzing large amounts of genomic information about metabolic networks. Similar weighted graphs could also be applied to other types of network: regulatory genetic, protein interaction, etc. The definitions of taxonomic and sequence similarity weighted graphs are directly applicable to other regulatory genetic and protein interaction graphs, and the definition of isoenzymatic weighted graphs could be adapted to be isofunctional. This would open up an interesting range of types of analysis for application to large numbers of genomes.

## Supporting Information

Table S1 The list of removed compounds taken from the article “Using a Logical Model to Predict the Growth of Yeast,” authored by K. E. Whelan and R. D. King, published in BMC Bioinformatics in 2008.
(PDF)
