# Correction: The Use of Weighted Graphs for Large-Scale Genome Analysis

**DOI:** 10.1371/journal.pone.0116432

**Published:** 2014-12-19

**Authors:** The PLOS ONE Staff

Figure 2 is erroneously cropped. Please view a complete version here.

**Figure 2:**
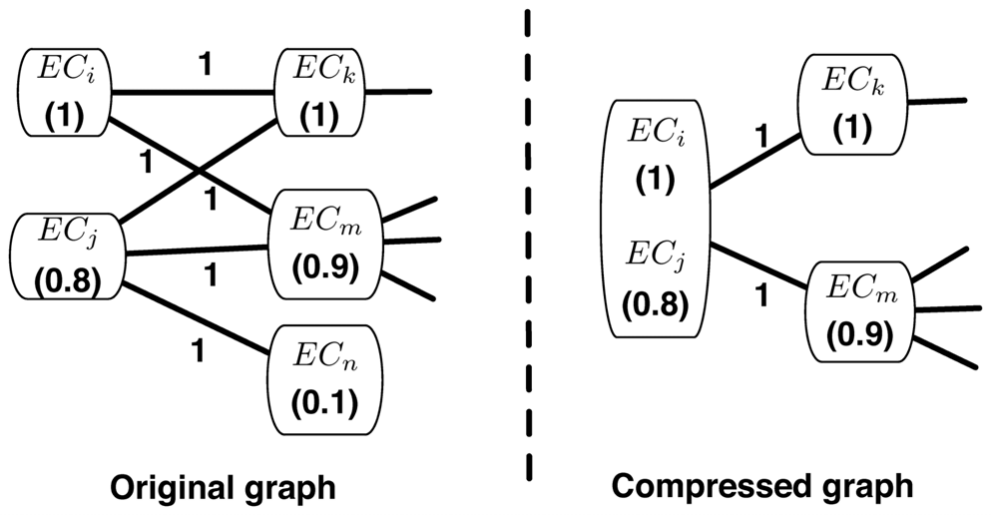
A example of weighted graph compression based on node weights. Node weights are in the parenthesis.
