# Supplementary material for: The Use of Weighted Graphs for Large-Scale Genome Analysis
Source: PLoS One. 2014 Mar 11;9(3):e89618. doi: 10.1371/journal.pone.0089618 (PMC3949676; doi:10.1371/journal.pone.0089618)
Supplement: Table S1 — The list of removed compounds taken from the article “Using a Logical Model to Predict the Growth of Yeast,” authored by K. E. Whelan and R. D. King, published in BMC Bioinformatics in 2008. (PDF) [file pone.0089618.s001.pdf]

Table S1: The list of the removed compounds taken from the article "Using a Logical Model to Predict the Growth of Yeast", authored by KE Whelan and RD King, published in BMC Bioinformatics in 2008.

Compound entry in KEGG	Compound name
C00001	H2O
C00002	ATP
C00003	NAD+
C00004	NADH
C00005	NADPH
C00006	NADP+
C00007	Oxygen
C00008	ADP
C00009	Orthophosphate
C00010	CoA
C00011	CO2
C00012	Peptide
C00013	Diphosphate
C00014	NH3
C00017	Protein
C00020	AMP
C00023	Iron
C00027	Hydrogenperoxide
C00028	Acceptor
C00030	Reducedacceptor
C00032	Heme
C00034	Manganese
C00038	Zinccation
C00050	Metal
C00070	Copper
C00076	Calciumcation
C00080	H+
C00087	Sulfur
C00088	Nitrite
C00138	Reducedferredoxin
C00139	Oxidizedferredoxin
C00237	CO
C00238	Potassiumcation
C00282	Hydrogen
C00291	Nickel
C00305	Magnesiumcation
C00697	Nitrogen
C00698	Cl-
C00703	Mercury(2+)
C00704	O2.-
C01319	Hg
C01328	HO-
C01342	NH4+
